# Impact of dark septate endophytes on salt stress alleviation of tomato plants

**DOI:** 10.3389/fmicb.2023.1124879

**Published:** 2023-06-21

**Authors:** Dalia A. Gaber, Charlotte Berthelot, Damien Blaudez, Gábor M. Kovács, Philipp Franken

**Affiliations:** ^1^Leibniz-Institute of Vegetable and Ornamental Crops, Grossbeeren, Germany; ^2^Erfurt Research Centre for Horticultural Crops, University of Applied Sciences, Erfurt, Germany; ^3^Department of Botany and Microbiology, Faculty of Science, Assiut University, Assiut, Egypt; ^4^Université de Lorraine, CNRS, LIEC, Nancy, France; ^5^CTIFL, Centre de Carquefou, Carquefou, France; ^6^Department of Plant Anatomy, Institute of Biology, Eötvös Loránd University, Budapest, Hungary; ^7^Centre for Agricultural Research, Plant Protection Institute, Budapest, Hungary; ^8^Institute of Microbiology, Friedrich Schiller University Jena, Jena, Germany

**Keywords:** dark septate endophytes, *Periconia macrospinosa*, *Cadophora* sp., *Leptodontidium* sp., albino mutant, salt stress alleviation, plant nutrition

## Abstract

Fungal endophytes can improve plant tolerance to abiotic stress conditions. Dark septate endophytes (DSEs) belong to phylogenetically non-related groups of root colonizing fungi among the Ascomycota with high melanin-producing activities. They can be isolated from roots of more than 600 plant species in diverse ecosystems. Still the knowledge about their interaction with host plants and their contribution to stress alleviation is limited. The current work aimed to test the abilities of three DSEs (*Periconia macrospinosa*, *Cadophora* sp., *Leptodontidium* sp.) to alleviate moderate and high salt stress in tomato plants. By including an albino mutant, the role of melanin for the interaction with plants and salt stress alleviation could also be tested. *P. macrospinosa* and *Cadophora* sp. improved shoot and root growth 6 weeks after inoculation under moderate and high salt stress conditions. No matter how much salt stress was applied, macroelement (P, N, and C) contents were unaffected by DSE inoculation. The four tested DSE strains successfully colonized the roots of tomato, but the colonization level was clearly reduced in the albino mutant of *Leptodontidium* sp. Any difference in the effects on plant growth between the *Leptodontidium* sp. wild type strain and the albino mutant could, however, not be observed. These results show that particular DSEs are able to increase salt tolerance as they promote plant growth specifically under stress condition. Increased plant biomasses combined with stable nutrient contents resulted in higher P uptake in shoots of inoculated plants at moderate and high salt conditions and higher N uptake in the absence of salt stress in all inoculated plants, in *P. macrospinosa*-inoculated plants at moderate salt condition and in all inoculated plants except the albino mutants at high salt condition. In summary, melanin in DSEs seems to be important for the colonization process, but does not influence growth, nutrient uptake or salt tolerance of plants.

## Introduction

Abiotic stress is one of the most limitation factors that negatively affect plant development and production (Potters et al., 2007; Thakur et al., 2010). During their life cycle, plants are subjected to many abiotic stress factors. Salinity stress is a very important environmental factor that leads to reduction of plant growth (Yamaguchi and Blumwald, 2005; Shahbaz and Ashraf, 2013). Eight hundred million hectares of agricultural lands are impaired due to soil salinity all over the world (FAO, 2008). To diminish the adverse effects of salt stress on plants, many strategies can be used such as leaching excessive salinity, growing salt-tolerant plants in salt-stressed soils or inoculate plants with beneficial microbes (Al-Karaki et al., 2001; Li et al., 2017). Many studies reported that endophytic fungi can alleviate salt stress of their associated plant (Jogawat et al., 2013; Laxmi et al., 2016; Li et al., 2017; Zhang et al., 2019; Bouzouina et al., 2020). For instance, plants inoculated with arbuscular mycorrhizal fungi (AMF) were reported to contain lower levels of Na (Dixon et al., 1993; Sharifi et al., 2007; Zuccarini and Okurowska, 2008; Balliu et al., 2015; Sallaku et al., 2019; Wang et al., 2022). Furthermore, it was demonstrated that *Serendipita indica* increases salt tolerance of associated plants (Waller et al., 2005; Baltruschat et al., 2008; Bagheri et al., 2013; Balliu et al., 2015; Yun et al., 2018; Abdelaziz et al., 2019; Kord et al., 2019; Sallaku et al., 2019).

Dark septate endophytes (DSEs) are a sub-group of endophytic fungi that belong to Ascomycetes and are featured by their melanized and septate hyphae. They produce conidial as well as sterile hyphae that colonize roots intracellularly or intercellularly. Most terrestrial plants are colonized by DSEs (Jumpponen and Trappe, 1998; Mandyam and Jumpponen, 2005), and DSEs could be isolated from plants living in different ecosystems (Jumpponen and Trappe, 1998; Kovács and Szigetvari, 2002; Rodriguez et al., 2009; Sonjak et al., 2009; Alberton et al., 2010; Knapp et al., 2012). However, little attention has been paid to DSEs compared to other groups of plant root colonizers (e.g., the AMF). DSEs showed antagonistic (Fernando and Currah, 1996; Yu et al., 2001b; Yakti et al., 2018), neutral (Berthelot et al., 2016), and mutualistic relations with plants (Andrade-Linares et al., 2011a,b; Vergara et al., 2018; Harsonowati et al., 2020). A meta-analysis suggested mainly positive effects, especially when nitrogen nutrition of the plants is limited (Newsham, 2011).

Tomato is an economically important crop; its productivity and yield are adversely affected by salt stress. The production and consumption of tomato are permanently increasing (Gerszberg et al., 2015). Besides its economic value, tomato is a model in plant science as long as it has many advantages such as easy maintenance, simple diploid genetics, short generation time, and easiness of genetic transformation (Barone et al., 2008). Altogether this make tomato an excellent species for both basic and applied plant research (Ranjan et al., 2012). It has been reported that DSEs positively affected some parameters and fruit quality of tomato plants (Andrade-Linares et al., 2011b). Moreover, Yakti et al. (2018) showed that *Periconia macrospinosa* and *Cadophora* sp. improved shoot biomass of tomato plants when cultivated with inorganic fertilizers.

In the current study, we used three models of dark septate endophytes *Periconia macrospinosa* (DSE 2036), *Cadophora* sp. (DSE 1049), *Leptodontidium* sp. (Me07, melanized WT) and the corresponding mutant of *Leptodontidium* sp. (non-melanized *albino* mutant Δ1110) for testing the following hypotheses. Firstly, DSEs inoculation under control and two levels of salt stress able to improve tomato growth and to confer salt stress tolerance. Secondly, melanin has a positive role in the interaction of DSEs with tomato plants and salt stress alleviation. Finally, DSE inoculation impact nutrient uptake of the plant under control or salt conditions.

## Materials and methods

### Dark septate endophytes used in this study

In our study, three strains of DSEs were used. The strains *P. macrospinosa* (DSE 2036), *Cadophora* sp. (DSE 1049; Knapp et al., 2012, 2015) and *Leptodontidium* sp. (Me07; Berthelot et al., 2016; Knapp and Kovács, 2016). An albino mutant of *Leptodontidium* sp. (Δ1110) was also used. This mutant was derived from the Me07 strain and was previously obtained by T-DNA insertion mutagenesis followed by phenotypical screening (Berthelot et al., 2017). Further information about origins and classification of the DSEs strains can also be found in Gaber et al. (2020).

### Preparation of DSEs inocula

Dark septate endophyte inocula were prepared according to the method described by Likar and Regvar (2013). Briefly, glass jars were filled with a mixture of 500 g vermiculite (RIGK GmbH, Wiesbaden, Germany) and 250 mL of potato dextrose broth (PDB; Roth, Karlsruhe, Germany) inoculated with seven plugs with 9 mm diameter of DSEs that were previously grown on potato dextrose agar (PDA) and Pachlewski agar media. Jars for mock inoculation received autoclaved plugs and media. These pots were sealed and incubated at 25°C in the dark for 3 weeks. During this incubation period, pots were shaken twice a week to enable homogenous growth of DSEs in the substrate.

### Plants–DSEs interaction experimental setup

To investigate the effect of DSEs on tomato growth and nutrient uptake, *Solanum lycopersicum* cv. Moneymaker was inoculated with the different DSE strains. Seedlings of tomato plants were inoculated with *P. macrospinosa*, *Cadophora* sp., *Leptodontidium* sp. and mutant Δ1110. The substrate for seed germination and plant growth after inoculation contained a 1:1 ratio of sand (particle size: 0.5–1 mm; Euroquarz, Ottendorf-Okrilla, Germany) and vermiculite (RIGK GmbH, Wiesbaden, Germany) and was autoclaved before introducing plants and inocula.

Plant seed surface sterilization was conducted by soaking seeds for 20 min in ethanol (70%), then 5 min in NaOCl and finally rinsed with distilled water. Seeds were germinated for 1 week before seedlings were transferred to the pots containing or not the DSE inocula. A preliminary plant experiment was established to define the appropriate salt levels for application in the main experiment. Different salt concentrations were tested to reach electric conductivities (EC) of 4, 6, 7, 8, 9, and 11 dS/m compared to control EC of 2.5 dS/m. In the main experiment, NaCl was added or not to Hoagland solution (De Kreij et al., 1997) to reach three different EC levels: 2.5 dS/m (non-salinized controls without NaCl addition), 5.5 dS/m corresponding to 60 mM of NaCl (moderate salt) and 10.5 dS/m corresponding to 115 mM of NaCl (high salt). Salinization was gradually increased until it reached the desired levels about 2 weeks after transplanting. The EC of the non-salinized control treatment (2.5 dS/m) was equal to the EC of the nutrient solution. Every treatment had 7 replicates in 7 plant pots. Plants were grown for 6 weeks after transplantation, inoculation and before flowering. Plants were grown under the following growth chamber conditions; 23/18°C at day/night (16 h/8 h), relative humidity value of 50% and light intensity of 400 μmol/m^−2^ s^−1^.

### Plant harvest and sample preparation for further analyses

At harvest, fresh weight (FW) of shoots and roots were recorded. Root aliquots were taken for staining by trypan blue (Phillips and Hayman, 1970; Koske and Gemma, 1989; Sigma-Aldrich, Munich, Germany) or wheat germ agglutinin—Alexa Fluor^™^ 488 (WGA-AF^™^ 488; Molecular Probes, Karlsruhe, Germany).

The remaining roots and shoots were dried for 2 days at 60°C, and dry weight (DW) was recorded. Shoot dry biomass was ground for further use in plant nutrient content analysis.

### Staining of fungal structures and root colonization intensity by DSEs

For confirmation of plant root colonization by DSEs, root fragments from each of the three plants per treatment were randomly chosen and stained by trypan blue (Phillips and Hayman, 1970; Koske and Gemma, 1989) and WGA-AF^™^ 488 (Deshmukh et al., 2007; Redkar et al., 2018). For trypan blue staining, 30 tomato root fragments of 10–15 mm length were treated with 5% KOH at 90°C for 15 min, then by 1% HCl at 20°C overnight. HCl was removed and replaced by 0.05% (w/v) trypan blue in lactoglycerol (1,1,1 lactic acid, glycerol and water), and heated for 30 min at 90°C. For discoloration, roots were incubated for 24 h in 50% (*v*/*v*) glycerol and stored in lactic acid at room temperature. Finally, colonization was monitored by light microscopy. For WGA-AF^™^ 488 staining, root fragments (5–10 mm length) were incubated with 5 μg mL^−1^ of WGA-AF^™^ 488 for 10 min. Fungal hyphae and structures were further visualized by confocal laser microscopy. The autofluorescence of root cells was detected between 420 and 470 nm. An argon laser was used to excite Alexa Fluor R 488, and fluorescence of fungal structures was detected between 500 and 550 nm. Images were obtained using an EVOS™ FL Cell Imaging System (Life Technologies, CA, United States).

The intensity of root colonization by DSEs was calculated based on 30 trypan blue-stained fragments (10–15 mm length) per sample according to previous studies (McGonigle et al., 1990; Likar and Regvar, 2013). Briefly, the calculation was based on a five-class system ranking: rare (*n*_1_; ~ 1% of the root fragment colonized), low (*n*_2_; 1–10%), medium (*n*_3_; 11–50%); high (*n*_4_; 51 90%); and abundant (*n*_5_; 91–100%).

Dark septate endophyte colonization intensity (%) = [(95x*n*_5_) + (70x*n*_4_) + (30x*n*_3_) + (5x*n*_2_) + (x*n*_1_)] / total number of fragments (30).

*n*_X_ = number of fragments rated as class X.

### Determination of shoot mineral element contents

Total phosphorus (P), nitrogen (N), carbon (C), sodium (Na) and potassium (K) contents were determined in plants (Gericke and Kurmies, 1952). Shortly, 200–300 mg of dried powder of shoots were digested in 5 mL 65% HNO_3_ and 2 mL of 30% H_2_O_2_ at 200°C for 15 min in a microwave (MARSXpress 250/50; CEM Corporation, North Carolina, United States). Distilled water was added to the digested samples to reach a volume of 25 mL. After filtration, P concentrations were obtained using a colorimetric spectrophotometer (EPOS 5060 analyzer, Eppendorf, Germany) at the wavelength 436 nm (Gericke and Kurmies, 1952). For N and C quantification, the filtrate was analyzed in an elemental analyzer (Elementar Vario EL, Elementar, Germany) according to DUMAS method (Dumas, 1831). Na and K concentrations were determined in the filtrate by emission spectroscopy with an emission spectrophotometer (ICP-OES, Thermo Fisher Dreieich, Germany) by using wavelengths of 589 and 592 nm for Na and 766 and 490 nm for K (Gericke and Kurmies, 1952). Inocula preparation, plant experiments and post-harvest analyses were carried out in laboratories and phytochambers of Leibniz Institute of Vegetable and Ornamental Crops (IGZ), Germany.

### Statistical analysis

All statistical analyses were carried out with the Statistica software (version 12, Tulsa, OK, United States). The normal distribution of data was inspected using the Kolmogorov–Smirnov test. Homogeneity of variance, as well as factorial analyses of variance (ANOVA) were conducted for detecting differences between values. Post-hoc analysis was carried out by Tukey HSD test.

## Results

### Root colonization by DSEs

**Figure 1:**
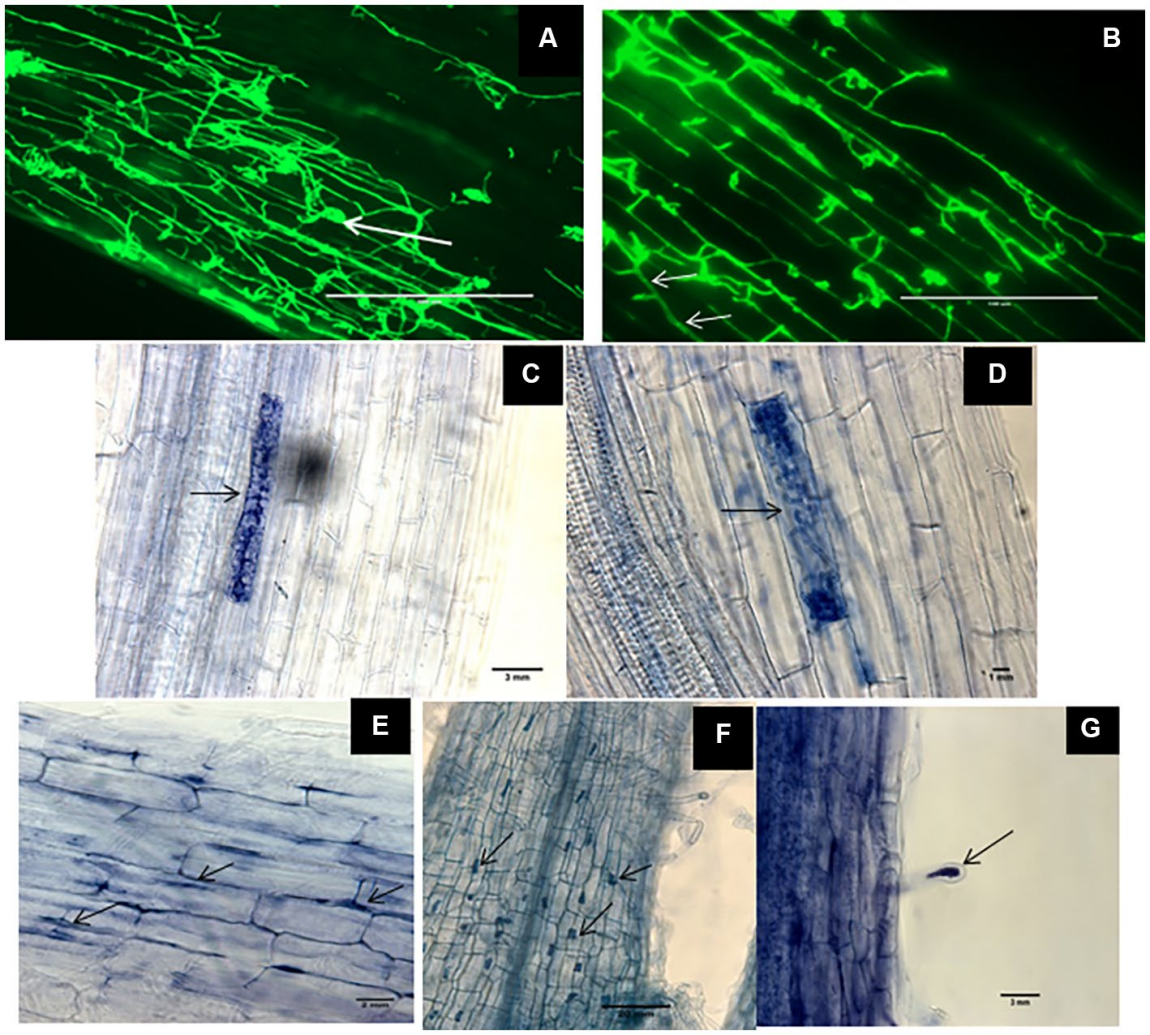
Morphological characteristics of Dark septate endophytes (DSEs) in tomato plant roots. WGA-AF^™^ 488 -stained root samples of tomato plants inoculated with *Periconia macrospinosa* and *Cadophora* sp. (**A,B**, respectively) were observed by confocal laser microscopy whereas trypan blue-stained roots and mycelia of *Leptodontidium* sp. were observed by optical microscopy **(C–G)**. Endophytic mycelia of *Periconia macrospinosa* **(A)** and aggregations of mycelia or microsclerotia-like structure (arrows in **A**). Septate mycelia *Cadophora* sp. (arrows in **B**) and intracellular hyphae of *Leptodontidium* sp. (arrows in **E**). Early developmental stage of microsclerotia **(C)** and loosely packed microsclerotia **(D)** of *Leptodontidium* sp. Microsclerotia-like structures of *Leptodontidium* sp. in root cells (arrows in **F**) and root hairs **(G)**.

Root samples of DSE-inoculated and mock-inoculated plants were stained with trypan blue and WGA-AF^™^ 488 to visualize the different fungal structures formed by the DSEs. No structures of DSEs could be observed in the roots of mock-inoculated plants. Inoculation with the three different DSEs and the melanin mutant did not cause any obvious disease symptoms. Microscopic observations of fungal structures in root samples confirmed successful DSE colonization of all inoculated plants. This was indicated by different particular morphological structures of DSEs inside plant roots. The roots of inoculated plants harbored intercellular hyphae of *P. macrospinosa* and *Cadophora* sp. (Figures 1A,B, respectively) and intracellular hyphae in *Cadophora* sp. and *Leptodontidium* sp. wild-type (WT; Figures 1B,E, respectively). As particular structures, characteristic septate hyphae of *Cadophora* sp. (Figure 1B), early developmental stage microsclerotia (Figure 1C) and microsclerotia of *Leptodontidium* sp. WT formed as a result of loosely packed cells (Figure 1D) were observed. Moreover, other structures were noticed such as microsclerotia-like structures of *Leptodontidium* sp. WT in both cortex and root hair cells (Figures 1F,G).

**Figure 2:**
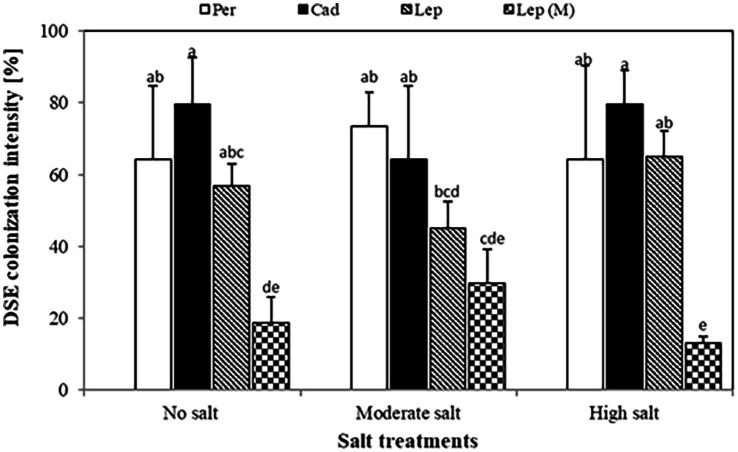
Dark septate endophyte (DSE) colonization intensity in tomato roots. Tomato plants were mock-inoculated (no fungus), inoculated with *Periconia macrospinosa* (Per), *Cadophora* sp. (Cad), *Leptodontidium* sp. WT (Lep) or *Leptodontidium* sp. mutant Δ 1110 (Lep (M)). All plants were grown in no salt, moderate salt (60 mM NaCl) or high salt (115 mM NaCl) substrates. There are significant interactions according to two-way ANOVA (*p =* 0.05, *n =* 7) between the factor salt level and DSEs. Different letters indicate significant differences obtained by Tukey HSD (*p* < 0.05).

Quantification of colonization revealed that colonization intensity of plants inoculated with *P. macrospinosa*, *Cadophora* sp., and *Leptodontidium* sp. WT ranged from 45.2 to 79.5% (Figure 2). Colonization intensity of plants inoculated with the *Leptodontidium* sp. mutant was significantly lower compared to that of plants inoculated with the WT strain. Interestingly, colonization was not affected by the increased levels of salt stress neither in the case of the wild type strains nor in the case of the albino mutant.

### Effect of DSEs on plant growth

**Figure 3:**
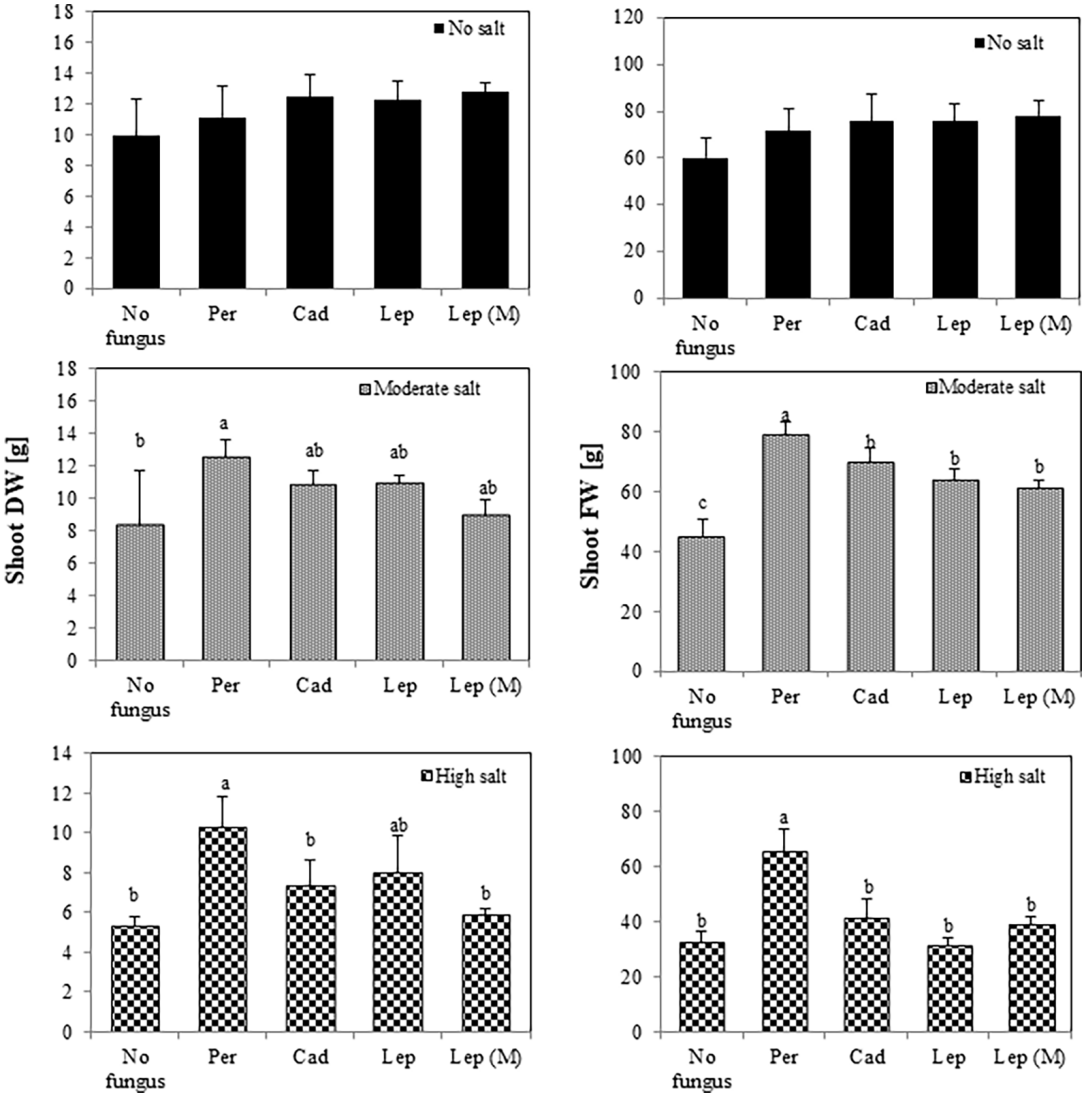
Impact of DSEs on tomato shoot biomass. Plants were mock-inoculated (no fungus), inoculated with *P. macrospinosa* (Per), *Cadophora* sp. (Cad), *Leptodontidium* sp. WT (Lep) or *Leptodontidium* sp. mutant Δ 1110 (Lep (M)) and were grown in no salt, moderate salt (60 mM NaCl) or high salt (115 mM NaCl) substrates. Fresh and dry weights of shoots were measured 6 weeks after inoculation. Two-way ANOVA (*p =* 0.05, *n =* 7) was carried out and results are shown in Supplementary Table S1. Significant differences between inoculated and non-inoculated plants are indicated by different letters (*p* < 0.05).

To investigate the effect of DSEs on plant growth, tomato plants were inoculated with different DSE isolates and FW and DW of the plants were measured 6 weeks after inoculation. Generally, the salt treatments dramatically reduced shoot and root FW (from 25.9 at no salt conditions to 8.9 gram at high salt conditions) and DW (from 4.4 at no salt conditions to 0.9 g at high salt conditions) of inoculated and mock-inoculated plants (no fungus) in comparison to the treatment without salt. After 6 weeks (Figure 3), none of the strains showed a significant influence on shoot FW or DW in the absence of salt stress. At moderate salt conditions, shoot FW was increased in all inoculations, but DW was only increased by *P. macrospinosa* (12.5 g) compared to control (8.28 g). *P. macrospinosa* was also the only fungus, which increased shoot FW (65.58 g) and DW (10.28 g) at high salt conditions compared to control plants with no DSE-inoculation (32.53 and 5.30 g, respectively; Figure 3).

**Figure 4:**
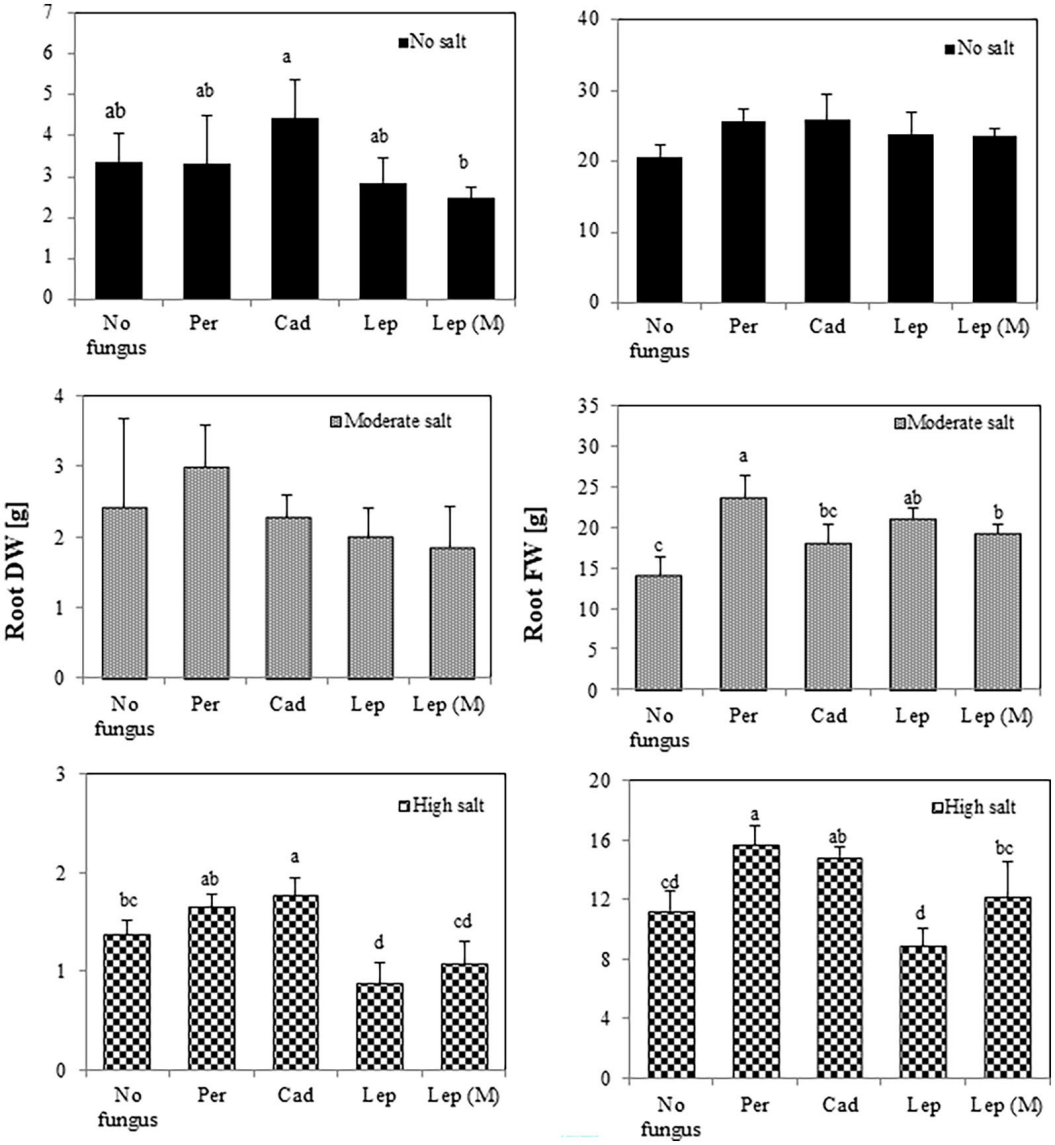
Impact of DSEs on tomato root biomass. Plants were non-inoculated (no fungus), inoculated with *P. macrospinosa* (Per), *Cadophora* sp. (Cad), *Leptodontidium* sp. WT (Lep) or *Leptodontidium* sp. mutant Δ1110 (Lep (M)) and were grown in no salt, moderate salt (60 mM NaCl) or high salt (115 mM NaCl) substrates. Fresh and dry weights of roots were measured 6 weeks after inoculation. Two-way ANOVA (*p* = 0.05, *n* = 7) was carried out and results are shown in Supplementary Table S1. Significant differences between inoculated and non-inoculated plants are indicated by different letters (*p* < 0.05).

Regarding root biomass (Figure 4), there was no effect of the strains on FW and DW in the absence of salt stress. There was, however, a significant increase of root FW in plants inoculated with *P. macrospinosa* (23.76 g), *Leptodontidium* sp. WT (21.15 g) and mutant Δ1110 (19.23 g) compared to controls (14.08 g) under moderate salt conditions. Under high salt conditions, *P. macrospinosa* (15.67 g) and *Cadophora* sp. (14.83 g) significantly improved root FW compared to control (14.08 g), while only plants inoculated with *Cadophora* sp. showed significantly higher root DW (1.78 g) compared to control (1.38 g). In contrast, *Leptodontidium* sp. WT significantly reduced root DW at high salt conditions compared to control conditions.

### Effect of DSEs on plant nutrition

In order to understand the contribution of DSEs in tomato acquisition of nutrients and how this could contribute to salt stress tolerance of plants, N, P, C, Na, and K contents were measured in shoots at the various salt-stress conditions.

**Figure 5:**
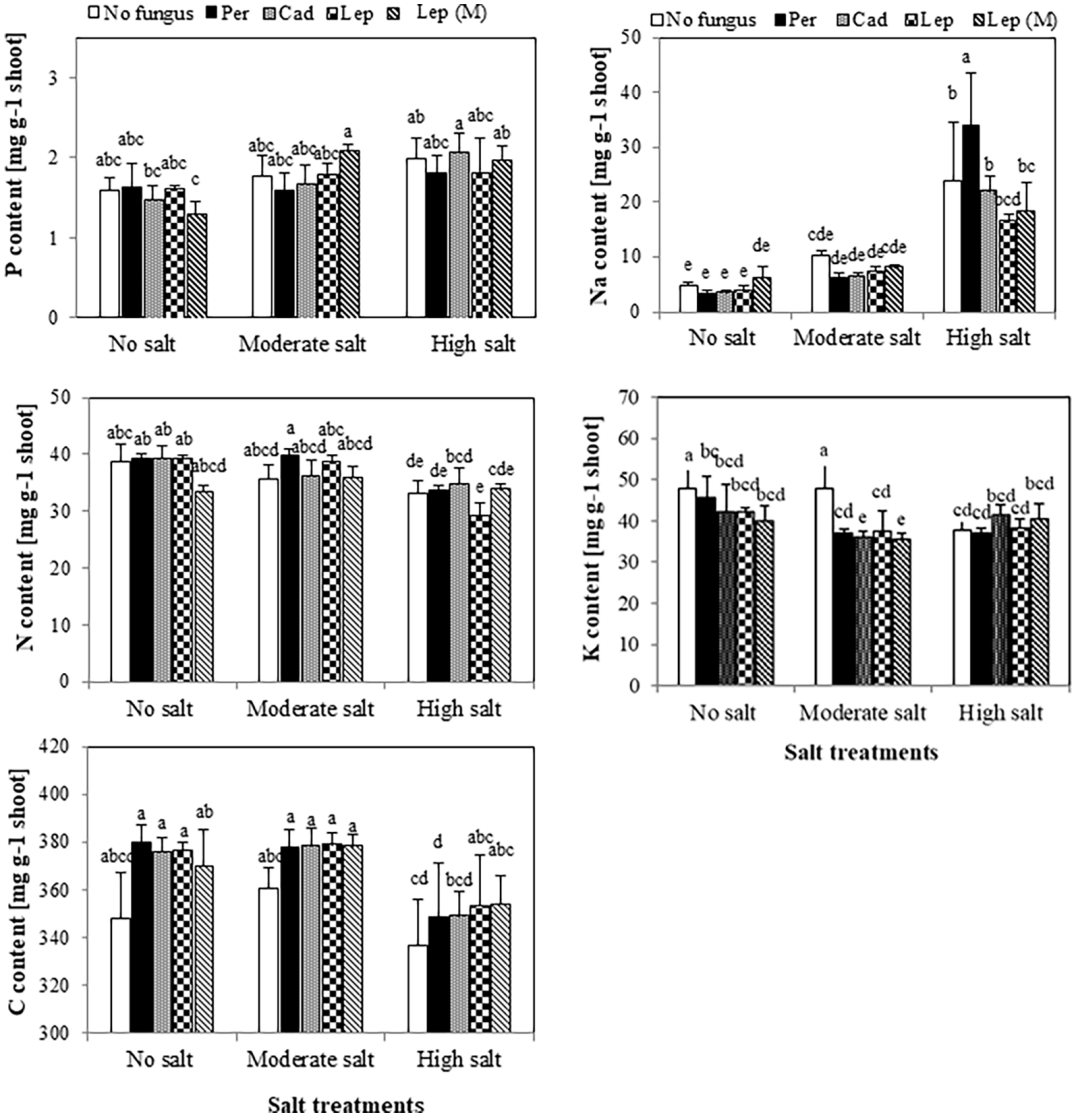
Impact of DSEs on the content of phosphorus (P), nitrogen (N), carbon (C), sodium (Na) and potassium (K) in tomato shoots. Plants were mock-inoculated (no fungus), inoculated with *P. macrospinosa* (Per), *Cadophora* sp. (Cad), *Leptodontidium* sp. WT (Lep) or *Leptodontidium* sp. mutant Δ1110 (Lep (M)) and were grown in no salt, moderate salt (60 mM NaCl) or high salt (115 mM NaCl) substrates. Contents of elements were measured. Two-way ANOVA (*p* = 0.05, *n* = 7) was carried out showing that the factors ‘DSEs’ and ‘salt’ had a significant impact on the concentration level of elements and that there was interaction between both factors for all elements. Significant differences are indicated by different letters (*p* < 0.05).

**Figure 6:**
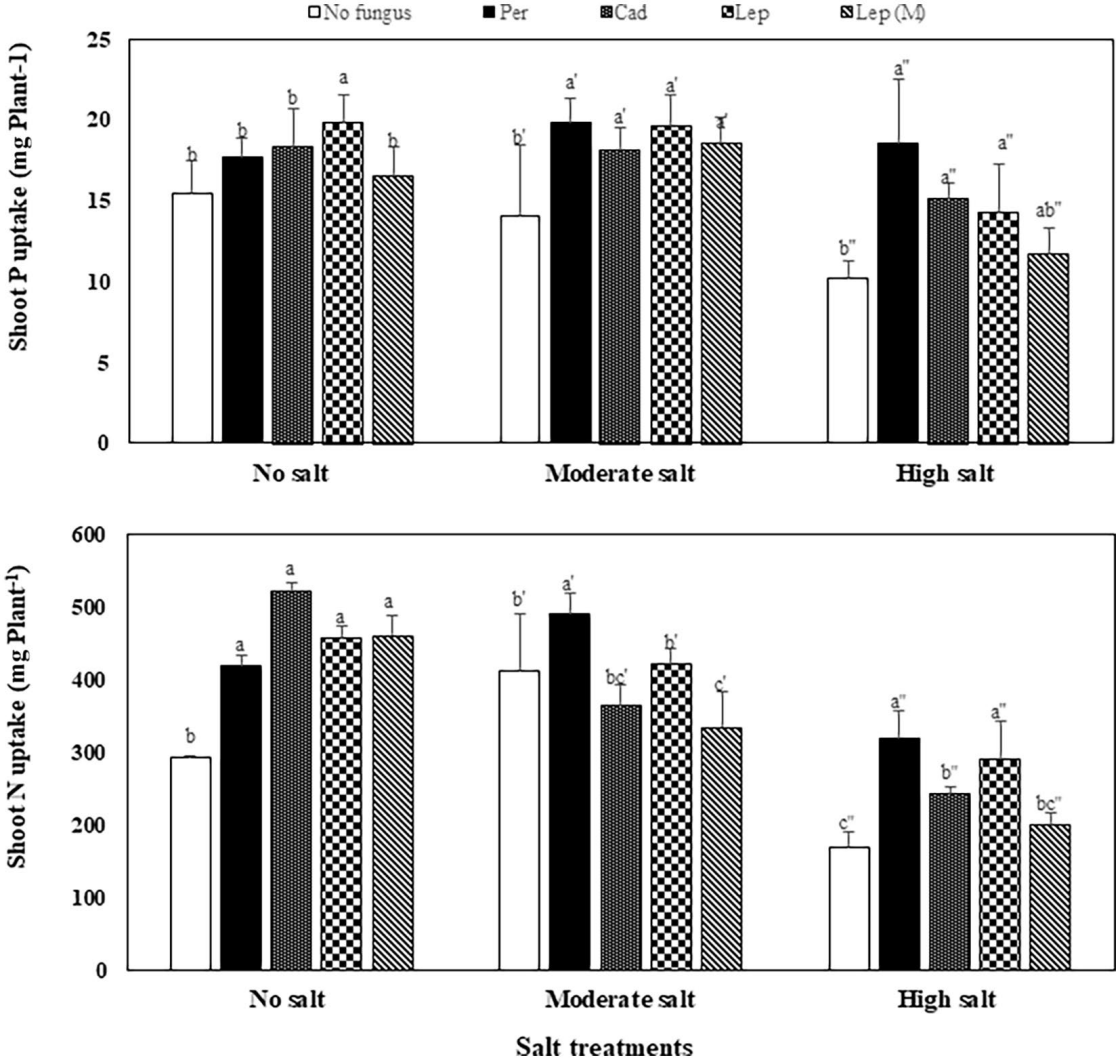
Impact of DSEs on the uptake of phosphorus (P) and nitrogen (N) in tomato shoots. Plants were mock-inoculated (no fungus), inoculated with *P. macrospinosa* (Per), *Cadophora* sp. (Cad), *Leptodontidium* sp. WT (Lep) or *Leptodontidium* sp. Mutant Δ1110 (Lep (M)) and were grown in no salt, moderate salt (60 mM NaCl) or high salt (115 mM NaCl) substrates. Two-way ANOVA (*p* = 0.05, *n* = 7) was carried out showing that the factors ‘DSEs’ and ‘salt’ had a significant impact on nutrient uptake and that there was interaction between both factors for all elements. Significant differences are indicated by different letters (*p* < 0.05).

The contents of P, N and C in shoots of plants that were inoculated with DSEs were not significantly different compared to mock-inoculated plants under all salt treatments (Figure 5). Similar trends were observed for Na contents in plants under all treatments apart from plants that were inoculated with *P. macrospinosa*. In this case, significantly higher Na contents in plant shoots (34 mg per g dry shoot weight) compared to mock-inoculated plants (24 mg per g dry weight shoot) were detected under high salt conditions (Figure 5). Inoculation of plants with DSEs could significantly decrease K contents compared to mock-inoculated plants under no and moderate salt conditions (Figure 5). Taking into account the DSE influence on biomass (Figure 6), P uptake per plant was significantly increased at moderate and high salt conditions by plant inoculation with all DSEs except *Leptodontidium* sp. Δ1110 mutant at high salt conditions. N uptake per plant was increased significantly when plants were inoculated with all DSEs in the absence of salt stress, *P. macrospinosa* at the moderate salt condition and with *P. macrospinosa*, *Cadophora* sp., *Leptodontidium* sp. at the high salt condition (Figure 6).

## Discussion

The impact of inoculation with the DSEs *P. macrospinosa*, *Cadophora* sp., *Leptodontidium* sp. and the melanin synthesis mutant of *Leptodontidium* sp. on tomato plants under control, moderate salt and high salt conditions was measured. Six weeks after inoculation of tomato plants, DSEs could successfully colonize plant roots as septate hyphae and microsclerotia of DSEs were visualized in roots. Microsclerotia and microsclerotia-like structures were detected in DSE-inoculated tomato roots. Microsclerotia have thick walls and can distinguished by their richness in glycogen, proteins and polyphosphates (Currah and Tsuneda, 1993; Yu et al., 2001a). Although, there is no information about the function and role of microsclerotia in plant colonization, Yu et al. (2001b) and Grünig et al. (2004) suggested that they may serve as propagules because their characteristics allow them to survive in living roots as well as in root debris. Moreover, it was previously reported (Berthelot et al., 2018) that there was no correlation between microscopy quantification of microsclerotia and the qPCR quantification of DSEs in plant roots. Probably, microsclerotia formation requires particular circumstances. For instance, carbon to nitrogen ratio of 10:1 for *Trichoderma asperellum* BRM-29104 strain that has ubiquitous ability to promote plant growth and health is the optimum nutrition conditions for microsclerotia high yield production (Locatelli et al., 2022). It could be similar in DSEs that microsclerotia formation needs special nutritional status such as high carbon content in the substrate. More studies are required to clarify the requirements needed for microsclerotia formation by DSEs in plant roots. Root colonization by DSEs has been reported to cause a variety of host responses (Jumpponen and Trappe, 1998). *Salix glauca* and *Asparagus officinalis* were, e.g., colonized by the DSEs *L. orchidicola* and *Phialocephala fortinii*, respectively, and stele tissue necrosis was observed in *Salix* roots (Fernando and Currah, 1996; Yu et al., 2001a). In the same context, Li et al. (2018) showed that some strains of *Paraconiothyrium* spp. and *Darksidea* spp. had a negative influence on the growth and mineral content of *Ammopiptanthus mongolicus*. In the current experiment, DSEs were able to colonize tomato roots without causing any disease symptoms, and this is in accordance with the results of Andrade-Linares et al. (2011a), Mahmoud and Narisawa (2013) and (Vergara et al., 2017), where DSEs also colonized tomato plants without pathogenicity symptoms.

It has been shown that DSEs could enhance tomato tolerance against different abiotic stress factors such as heavy metals. For instance, DSE strains of *Phialophora mustea* improved tomato seedlings growth and enhanced Zn and Cd tolerance by the reduction of metal uptake into roots and shoots and the improvement of antioxidant enzymes activities (Zhu et al., 2018). The effect of DSEs on plant growth under salt conditions has been rarely investigated. Here, DSEs could improve shoot and root growth 6 weeks after inoculation of tomato plants with *P. macrospinosa* and *Cadophora* sp., tomato shoot and root growth were enhanced under moderate and high salt conditions. Similarly, our data showed that *P. macrospinosa* and *Cadophora* sp. have induced growth promotion of tomato plants under control and different salt conditions. We showed that *Leptodontidium* sp. showed mostly neutral effect on tomato plant growth under control and salt conditions. In accordance with this result, some reports (Berthelot et al., 2016, 2018) showed that plants inoculated with DSEs did not show any effect on plant growth. As *P. macrospinosa* was able to confer salt stress tolerance, the first hypothesis therefore is accepted.

Under saline conditions, Cofré et al. (2012) showed that roots of *Atriplex cordobensis* colonization by DSEs did not show significant differences when isolated from three different saline sites indicating that DSEs could confer salt stress tolerance to plants. Furthermore, DSE positively contributed in the root development of host plants and alteration of the soil nutrient content and microbiota under different NaCl concentrations used (0, 1, 2, 3 g NaCl/kg soil; Hou et al., 2021). The inoculation of *Phragmites australisa* roots with DSE GG2D improved the survival of plant seedlings under salt stress (Gonzalez Mateu et al., 2020). Likewise, our data show that the intensity of root colonization by DSEs was not impacted by salt stress. Pan et al. (2018) also reported that DSEs had positive influences on the root morphological characteristics under salt-stress conditions. We previously reported the high level of salt stress tolerance of the DSEs used in our present study (Gaber et al., 2020). This therefore could explain the high root colonization intensity found under salt conditions. In addition, Macia-Vicente et al. (2012) showed that *Lophiostoma* sp. isolate with coding no. OTU01 was a highly colonizer of roots of *Inula crithmoides*, a halophytic plant, in the lower salt marsh.

As in AMF, there is not always a correlation between plant growth and the root colonization intensity by DSEs (Hildebrandt et al., 2007; Regvar et al., 2010). Results showed that tomato plants were less colonized by the albino mutant compared to the WT of *Leptodontidium* sp. and this was positively correlated with improved plant growth. The mechanisms underlying plant roots colonization by DSEs are rarely investigated. In this regard, Fernández et al. (2013) observed that the initial colonization of *Megalastrum spectabile* and *Blechnum magellanicum* roots consisted in superficial narrow, septate and more frequently melanized runner hyphae. Then, at the point where DSE-root penetration into the cortical cells, different types of melanized appressoria (swollen structures preceding penetration) were detected (Andrade-Linares et al., 2011b). Appressoria, which were initially discovered in plant pathogens, are also found in epiphytes, endophytes, saprobes, entomopathogens, and symbionts (Emmett and Parbery, 1975; Capital and Lao, 2020). Appressoria are therefore not organs that have developed particularly for plant pathogens infection mechanisms. When this information is linked with our findings, it suggests that melanin plays a key function in root colonization by DSEs. Although the current results gave already first hints for the impact of melanin accumulation on the interaction with the plant, this points still to the need for further experiments to investigate the mechanisms of melanin contribution in plant roots colonization and development of DSEs hyphal structures in root cells. Our second hypothesis therefore could be accepted.

Dark septate endophytes can improve tomato plant growth under different conditions. Andrade-Linares et al. (2011a) reported such growth promotion by the DSE fungus *L. orchidicola* under optimal mineral inorganic nutrient conditions (Mahmoud and Narisawa, 2013; Vergara et al., 2017) showed such effects in the presence of organic N. The capacity of DSEs to produce phytohormones is one of the suggested mechanisms for enhancing growth of plants. Several studies have found that endophytic fungi (such as *Chaetomium globosum*, *Phoma glomerata*, *Penicillium* sp., and *Serendipita indica*) produce indole acetic acid (IAA; Sirrenberg et al., 2007; Khan et al., 2011). IAA synthesis by endophytic fungi may play important roles in root and shoot growth and improve root surface absorption area that resulting to higher water and nutrient uptake and facilitate root colonization by other fungi (Sukumar et al., 2013).

Our results confirm that DSEs could enhance tomato plant growth in the presence of inorganic N. Many previous studies have discussed the role of DSEs in plant nutrition as they improve not only the growth, but also the nutrient content of inoculated plants (Haselwandter and Read, 1982; Jumpponen and Trappe, 1998). In addition, Knapp and Kovács (2016) confirmed that *P. macrospinosa* and *Cadophora* sp. have a broad capacity of enzymatic activities that enable them to use different sources for nutrition. In our study, tomato plants inoculated with DSEs exhibited higher dry weights of the shoots and roots than non-inoculated control plants in some treatments. This resulted in a significant increase in P and N uptake per plant, although the P contents did not differ between DSEs-inoculated and control plants whatever the experimental condition with or without salt stress. Therefore, the third hypothesis that nutrient uptake can be enhanced by DSEs can be accepted. This increased uptake leads to increased growth without the nutrient content is changed.

Physiologically, plants apply a strategy to cope with salt stress by maintaining ionic homeostasis (Zhu, 2001; Hu et al., 2012) because the accumulation of Na in high amounts is highly toxic for plants (Fortmeier and Schubert, 1995; Kohler et al., 2009). In addition, excessive amounts of Na lead to minimize K contents which finally cause stomatal disturbance. Moreover, high Na accumulation induces oxidative stress and decline photosynthesis (Mahajan and Tuteja, 2005). On the other hand, K activate many important enzymes in plant metabolism (Bhandal and Malik, 1988; Wang et al., 2013). Our results showed that plants inoculation with DSEs did not impact neither Na except in the plants inoculated with *Periconia macrospinosa* nor K contents in shoots. In accordance with our results, Farias et al. (2020) showed that salinity caused increase in calcium, sodium, and chloride contents in cowpea plants regardless of inoculation with DSEs (*Sordariomycetes* sp. 1-B′2 and *Melanconiella elegans*-21 W2). We suggest that the mechanism of alleviation of salt stress on plants by DSEs does not depend on balancing K/Na ratios.

It has been reported in many studies that melanin possesses antioxidant activities acting as a scavenger of reactive oxygen species produced in excessive amounts under stress conditions (Dharmik and Gomashe, 2013; Sun et al., 2017; Łopusiewicz et al., 2018; Łopusiewicz, 2018). In our study, we found that only tomato plants inoculated with the *Leptodontidium* sp. albino mutant Δ1110 showed higher shoot growth than mock-inoculated plants and plants inoculated with the WT under high salt conditions. We therefore reject this hypothesis and suggest that melanin does neither contribute to salt stress tolerance of the fungus itself (Gaber et al., 2020) nor does it play any role in plant growth improvement or salt stress alleviation in the plant.

## Data availability statement

The original contributions presented in the study are included in the article/Supplementary material, further inquiries can be directed to the corresponding authors.

## Author contributions

DG and PF designed the research, interpreted the data, and wrote the manuscript. DG performed the research and analyzed the data. GK, DB, and CB provided the endophytes under investigations and co-supervised the experimental work. All authors contributed to the article and approved the submitted version.

## Funding

DG was supported by a Yousef Jameel PhD grant provided by the Humboldt Universität zu Berlin. This project has received funding from the Ministry of Consumer Protection, Food and Agriculture of the Federal Republic of Germany, from the Ministry for Science, Research and Culture of the State of Brandenburg, and from the Thuringian Ministry of Infrastructure and Agriculture.

## Conflict of interest

The authors declare that the research was conducted in the absence of any commercial or financial relationships that could be construed as a potential conflict of interest.

## Publisher’s note

All claims expressed in this article are solely those of the authors and do not necessarily represent those of their affiliated organizations, or those of the publisher, the editors and the reviewers. Any product that may be evaluated in this article, or claim that may be made by its manufacturer, is not guaranteed or endorsed by the publisher.
