# Supplementary material for: Impact of dark septate endophytes on salt stress alleviation of tomato plants
Source: Front Microbiol. 2023 Jun 21;14:1124879. doi: 10.3389/fmicb.2023.1124879 (PMC10320394; doi:10.3389/fmicb.2023.1124879)
Supplement: Supplementary file 1 [file Table_1.docx]

**Table 1.S.: Effect of DSEs inoculation and salt stress interaction on tomato plant growth under different salt stress levels**

Supplementary files

| **Growth**  **parameters** | | **No salt** | | | | | **Moderate salt** | | | | | **High salt** | | | | |
| --- | --- | --- | --- | --- | --- | --- | --- | --- | --- | --- | --- | --- | --- | --- | --- | --- |
|  |  | **No fungus** | **Per** | **Cad** | **Lep** | **Lep (M)** | **No fungus** | **Per** | **Cad** | **Lep** | **Lep (M)** | **No fungus** | **Per** | **Cad** | **Lep** | **Lep (M)** |
| **6 weeks after inoculation** | Shoot FW [g] | 60.2^bc^ ± 8.4 | 71.5^ab^ ± 9.7 | 75.6^ab^ ± 11.7 | 75.8^ab^ ± 7.4 | 78.0^a^ ± 6.3 | 45.2^cd^ ± 5.5 | 79.0^a^ ± 4.1 | 69.7^ab^ ± 4.7 | 64.1^ab^± 3.3 | 61.3^bc^ ± 2.2 | 32.5^e^ ±3.9 | 65.6^ab^ ± 7.9 | 41.3^e^ ± 6.8 | 31.1^e^± 3.0 | 38.9^e^ ± 8.4 |
|  | Shoot DW [g] | 10.0^ab^ ± 2.3 | 11.1^ab^ ± 2.1 | 12.5^a^ ± 1.4 | 12.3^a^ ± 1.3 | 12.8^a^ ± 0.6 | 8.3^bc^ ± 3.5 | 12.5^a^ ± 1.1 | 10.8^ab^ ± 0.9 | 10.9^ab^ ± 0.5 | 8.9^abc^ ± 1.0 | 5.3^d^ ± 1.4 | 10.3^ab^ ± 1.3 | 7.4^bc^ ± 0.7 | 8.0^bc^ ± 1.2 | 10.0^d^ ± 2.4 |
|  | Root FW [g] | 20.7^bc^ ± 1.5 | 25.7^a^ ± 1.7 | 25.9^a^  ± 3.5 | 24.0^ab^ ± 3.1 | 23.6^ab^ ± 1.0 | 14.1^ef^ ± 2.2 | 23.8^ab^ ± 2.6 | 18.2^cde^ ± 2.2 | 21.2^abc^± 1.1 | 19.3^bcd^ ± 1.0 | 11.2^fg^ ± 1.4 | 15.7^def^ ± 1.3 | 14.8^def^ ± 0.7 | 8.9^h^ ± 1.2 | 12.2^fg^ ± 2.4 |
|  | Root DW [g] | 3.4^bc^ ± 1.7 | 3.3^bc^ ± 1.2 | 4.4^a^ ± 1.0 | 2.9^bcde^± 0.6 | 2.5^cdef^ ± 0.3 | 2.4^cdef^ ± 1.3 | 3.0^bcd^ ± 0.6 | 2.3^cdef^ ± 0.3 | 2.0^cdef^± 0.4 | 1.9^cdef^ ± 0.6 | 1.4^ef^ ± 0.2 | 1.7^def^ ± 0.1 | 1.8^cdef^± 0.2 | 0.9^g^ ± 0.2 | 1.1^g^ ± 0.2 |

Plants were mock-inoculated (no fungus), inoculated with *P. macrospinosa* (Per), *Cadophora* sp. (Cad), *Leptodontidium* sp. WT (Lep) or *Leptodontidium* sp. Mutant Δ1110 (Lep (M)) and were grown in no salt, moderate salt (60 mM NaCl) or high salt (115 mM NaCl) substrates. Two-way ANOVA (*P* = 0.05, *n* = 7) was carried out showing that the factors ‘DSEs’ and ‘salt’ had a significant impact on nutrient uptake and that there was interaction between both factors for all elements. Significant differences are indicated by different letters (p<0.05).
